# Type 1 Diabetes Autoimmunity: To What Extent Vitamin D is Beneficial

**DOI:** 10.1155/bri/3557065

**Published:** 2025-12-04

**Authors:** Haitham Al-Madhagi

**Affiliations:** ^1^ Biochemical Technology Program, Dhamar University, Dhamar, Yemen

**Keywords:** autoimmune disorders, autoimmunity, clinical trials, Type 1 diabetes, vitamin D

## Abstract

Autoimmune disorders, characterized by an overactive immune system attacking healthy tissues, are a significant global health concern. A common thread among these disorders is vitamin D deficiency. While the exact relationship between vitamin D and autoimmunity remains a hot research arena, plenty of reports have suggested a strong association between vitamin D and autoimmune conditions, including systemic lupus erythematosus, rheumatoid arthritis, multiple sclerosis, and Type 1 diabetes, among others. Type 1 diabetes, in particular, has been extensively studied in relation to vitamin D. Evidence suggests that vitamin D deficiency may play a role in the development of this autoimmune disease. Beyond its well‐known function in bone health, vitamin D acts as a key regulator of both innate and adaptive immune responses. This review sought to explore the complex interplay between vitamin D and the immune system, with a particular focus on its potential role in autoimmunity, especially Type 1 diabetes. Moreover, the coupled therapy and clinical trials that have investigated the use of vitamin D as a therapeutic intervention for autoimmune disorders were also examined, highlighting both successes and limitations.

## 1. Introduction

The evolution of work has significantly altered the extent of human exposure to sunlight. From early agrarian societies, where daily labor was predominantly outdoors, to the rise of industrialization and the subsequent shift toward indoor occupations, the balance between sun exposure and work has dramatically changed. This transformation has implications for human health, particularly in relation to vitamin D synthesis and skin cancer risk [1].

Humans have a physiological need for sunlight, mainly in light of their ability to synthesize vitamin D through its action for various physiological functions. This is realized by outdoorsmen, who show significant relations with higher serum 25‐hydroxyvitamin D (25OHD) concentrations compared to indoorsmen. As such, for each hour in the sun during summer workdays, outdoor workers experienced a 5.2% increase in the levels of 25OHD [2, 3]. Outdoor occupation has also been linked to a reduced risk of neurodegenerative diseases, including Parkinson’s disease, an indication, without a doubt, that sunlight exposure does come with health benefits [4]. Although the recommendations of many medical associations have focused in the recent decades to avoid sunlight to lessen the risk of melanoma incidence, they completely ignore the downside of vitamin D status and requirements [5].

Sunlight is still a fundamental source of vitamin D in humans; 25OHD levels are exceptionally higher in outdoor workers. Increased outdoor exposure is associated with reduced risks of diseases such as Parkinson’s disease [6]. The deficiency in vitamin D may cause low serum calcium and phosphate levels, thus affecting the general health. A good extent of sunlight exposure is necessary to have the optimal level of vitamin D, which may be associated with musculoskeletal health [7].

The foundational discovery of vitamin D’s essential physiology emerged from its identification as the antirachitic factor, critical for systemic mineral ion regulation and the subsequent orchestration of bone mineralization. Recent research has expanded the understanding of vitamin D, showcasing the positive effects beyond skeletal tissues [8, 9]. Indeed, it is now clear that the roles vitamin D play in other tissues and organs shed light on the pathophysiology of autoimmune cases such as Type 1 diabetes (T1D), multiple sclerosis, rheumatoid arthritis, and inflammatory bowel syndrome [10].

T1D is a chronic metabolic disorder characterized by the autoimmune‐mediated destruction of insulin‐producing pancreatic β cells, leading to absolute insulin deficiency. The etiological foundation of T1D is firmly established as an organ‐specific autoimmune disease, a concept supported by a confluence of evidence including genetic susceptibility, the presence of autoantibodies, and insulitic lesions. The pathophysiology involves a loss of immunological self‐tolerance, triggering a targeted attack on β cells by autoreactive T lymphocytes [11]. However, the incomplete concordance of T1D in monozygotic twins (∼50%) highlights the essential role of environmental factors in triggering the autoimmune process in genetically susceptible individuals [12].

The exact pathways through which vitamin D influences these autoimmune conditions are yet to be elucidated. Albeit it is true that there is a clear correlation between vitamin D deficiency and autoimmunity, scientists are figuring out the type of this correlation, i.e., whether it is a cause or a complication.

This article sought to investigate the roles of vitamin D in immunity and autoimmunity, with an emphasis on T1D, to infer conclusive findings from the updated clinical trials and to identify areas that warrant further investigation. In addition, the coupling therapy of vitamin D with other medications to treat T1D is also discussed.

This narrative review introduces novelty by addressing a critical translational gap in the vitamin D and T1D field. While previous reviews [13, 14] have established the fundamental and epidemiological link between vitamin D and T1D, this work specifically identifies the unresolved mechanistic and clinical trials from updated data that hinder therapeutic application. A key and pioneering focus is its exploration of vitamin D not as a monotherapy, but within combination therapy regimens, proposing a new, clinically relevant paradigm for future T1D research and treatment strategies.

## 2. The Multifaceted Role of Vitamin D in the Body

**Figure 1:**
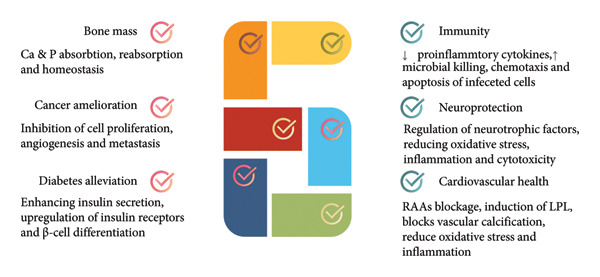
A wide spectrum of the different roles vitamin D plays in the body involving bone mass maintenance, immunity and cancer, neurological, and cardiovascular and diabetes prevention and alleviation.

Vitamin D is a fat‐soluble vitamin that plays an important role in fulfilling many physiological requirements of the human body. Its chief role and function are related to maintaining the normal level of calcium and phosphorus in the body, which are mainly responsible for bone and tooth health. However, the role of vitamin D is not confined to skeletal health [15], as depicted in Figure 1.

### 2.1. Bone Health and Mineralization

Vitamin D facilitates the intestinal absorption of calcium and maintains optimal serum calcium and phosphate concentrations, which are necessary for bone mineralization. Inadequate levels of vitamin D are responsible for the disorders of rickets in children and osteomalacia or osteoporosis in adults. Overall, all these conditions are characterized by weakened bones, increased risk of fractures, and loss of skeletal strength [16]. Studies found a positively significant association between bone mineral density and vitamin D status in menopausal women by using radiofrequency echographic multispectrometry technology [17]. In a Chinese study, vitamin D supplementation was accompanied by improvement (decrease) in lipid profile, fasting plasma glucose, and bone mass in pediatrics [18].

### 2.2. Regulation of the Immune System

Vitamin D has gained increasing interest regarding its contribution to immune function. It enhances the pathogen‐fighting properties of monocytes and macrophages, white blood cells necessary for the immune response. Appropriate levels of vitamin D relate to a reduced incidence of diseases involving infections and autoimmune disorders such as multiple sclerosis and rheumatoid arthritis. The immunomodulatory effect helps the body maintain a balanced immune response and avoid overactivity of the immune response that would lead to inflammation and tissue damage [19].

Randomized controlled trials (RCTs) in healthy older adults show that vitamin D3 supplementation (e.g., 1000 IU/day for 12 weeks) can increase lymphocyte subset counts and modulate inflammatory cytokines, indicating improved immune function [20].

Also, in critically ill COVID‐19 patients attending intensive care units, high‐dose vitamin D3 supplementation (5000 IU/day) showed increased natural killer (NK) and NK T (NKT) cells and improved immune profiles, indicating enhanced cellular immunity [21].

Even in clinical trials targeting autoimmune Graves, vitamin D supplementation (2800 IU) has been shown to reduce disease activity by rebalancing immune responses toward tolerance, decreasing proinflammatory cytokines, and suppressing autoantibody production, albeit authors did not recommend vitamin D for this condition [22].

### 2.3. Cardiovascular Health

An accumulated body of evidence is emerging that suggests the instrumental role for vitamin D in cardiovascular health. It is firmly linked to lower blood pressure, improved endothelial function, and slightly decreased inflammation, all of which are attributes that contribute to maintaining a healthy heart. Low levels of vitamin D have been linked to increased risk of heart disease; however, this is one of many factors to consider and requires further study [23]. A systemic analysis of 6 clinical trials for evaluating vitamin D against cardiovascular deficits found inconsistent results. It was figured out that the effects of vitamin D are temporal [24]. Also, vitamin D–statins drug interactions were found in those taking cholesterol‐lowering medications secondary to Type 2 diabetes [25].

### 2.4. Mental Health and Cognitive Function

Vitamin D is an important nutrient for the brain and has been implicated in the prevention of neurodegenerative diseases. In fact, there is an accumulated body of evidences that correlate the most common forms of dementia and neurodegeneration such as Alzheimer’s disease with insufficient vitamin D intake or exposure [26]. The receptor of vitamin D was shown to be widely distributed within the human brain and even on the surface of the immune glial cells, suggesting possible mechanisms of action for vitamin D in the brain, including neuroprotection, modulation of neuroinflammation, and support for synaptic plasticity. Indeed, numerous studies have found low serum vitamin D levels associated with poorer cognitive performance, including executive dysfunction and impairments in memory, particularly among older individuals [27]. Moreover, recent research has shown that higher levels of vitamin D in the brain relate to a lower risk of dementia development, which could mean that vitamin D offers protection against cognitive loss [28]. Supplementation has been studied as a route to treatment, but the specifics of how vitamin D impacts cognitive health remain an active area of inquiry. For example, in a clinical trial extended for 12 months, vitamin D supplementation was shown to greatly reduce oxidative stress, improve telomere shortening, and improve cognitive decline in elderly subjects with mild cognitive impairment (*p* < 0.001) [29].

### 2.5. Cancer Prevention

Vitamin D has been touted as a very important modality in the prevention and management of cancer, since it acts through varied biologic pathways which prevent tumor growth and its progression. Many forms of cancer involving cervical, prostate, and lung cancers were concomitantly associated with abnormally low blood vitamin D levels [30]. This can be interpreted by the fact that vitamin D exerts an antitumor activity through triggering apoptosis of cancerous cells, along with the blockage of their proliferation. This step facilitates the recognition as well as subsequent targeting by immune cells [31–33]. Studies have demonstrated an inverse association between the amount of vitamin D and cervical neoplasia; this states that the food substance provides protection in the initial phase of cervical cancer. Vitamin D is also seen to have positive effects on the clinical outcomes of ovarian and endometrial cancers, among gynecological malignancies [34]. Moreover, both cholecalciferol as well as calcitriol proved antitumor activity against breast and prostate cancers in animal models [35]. Also, some vitamin D3 derivatives exhibited antitumoral potency on glioblastoma cells. These effects were induced through arresting the cellular cycle process [36]. It can also instigate apoptosis of glioma cells of rats in vitro [37].

It is relevant to point out that vitamin D is an essential nutrient with multidiversified and major roles in the organism, from the health of bones to the modulation of immune response and mental state. Vitamin D levels should be appropriate through sun exposure, diet, and supplementation when necessary, for overall health maintenance and in the prevention of many diseases.

## 3. Biochemistry of Vitamin D

**Figure 2:**
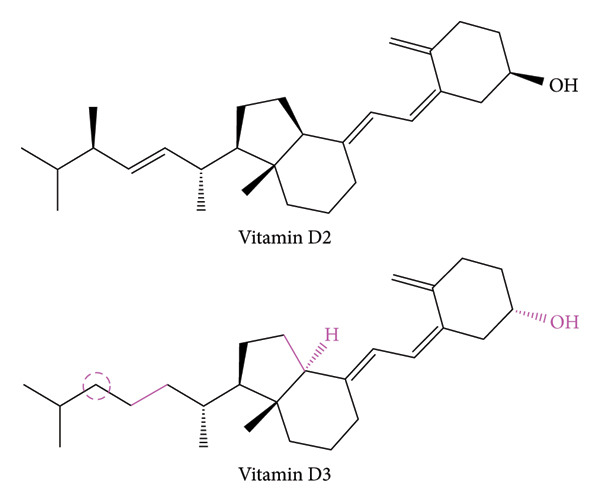
Structural comparison of vitamin D2 and vitamin D3. These include the orientation of functional groups and other bonds, in addition to the presence of extra methyl. Differences are marked in pink.

Mankind can acquire vitamin D in two major forms: plant‐based vitamin D2 (ergocalciferol) and sunlight‐based vitamin D3 (cholecalciferol). There is a slight chemical structural difference between the two forms as highlighted in pink in Figure 2. Such minute distinctions render vitamin D2 to have lower affinity to the vitamin D‐binding protein (DBP), which results in a faster rate of clearance out of the body and, thus, comparatively diminished bioavailability. Consequently, cholecalciferol (vitamin D3) is the predominant form of vitamin D [38].

Cholecalciferol has to be active and play the mentioned pivotal roles; it first has to be subjected to a series of photocatalytic as well as enzymic reactions. The journey begins with the UVB light of the sun, which converts 7‐dehyrocholestrol found beneath the skin into vitamin D3. It then moves to the liver where it acquires a hydroxyl moiety via 25‐hydroxylase (CYP2R1) to form hydroxycholecalciferol (25‐OHD3). Finally, 25‐OHD3 is transferred to the kidney to acquire another OH‐moiety catalyzed by 1α‐hydroxylase (CYP27B1), producing 1,25‐dihydroxycholecalciferol (1,25‐(OH)2D3, also known as calcitriol). The renal enzyme CYP27B1 has been shown to be also distributed into a variety of immune cells to perform autocrine and paracrine secretion and responses of calcitriol [39, 40].

**Figure 3:**
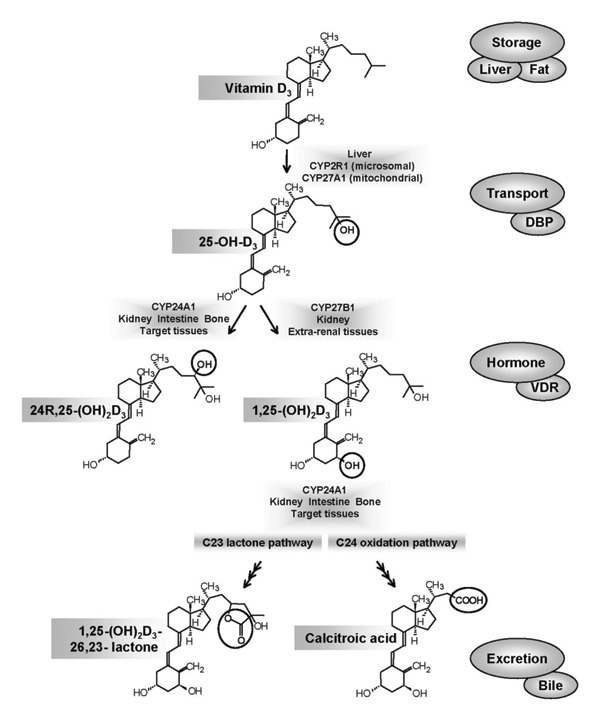
Overall metabolism of vitamin D3, including biosynthetic scheme, transport in blood, along with its active metabolites’ fate and excretion. Figure was reused from [40] under CC‐BY 4.0. Synthesis begins with the addition of an OH group in the 25C of vitamin D3 in the liver, followed by another hydroxylation in the kidney to form calcitriol. Its movement in the blood is assisted by vitamin D‐binding protein (DBP). Calcitriol has 2 degradative pathways: the C24 pathway forming calcitroic acid and the C23 lactone pathway forming 23, 26‐ lactonocalcitriol, which are ready to be excreted in bile.

In the kidney, the activity of CYP27B1 was found to be under control of certain hormones and growth factors including parathyroid hormone, Fibroblast growth factor 23, and calcitriol itself through a negative feedback mechanism. Conversely, CYP27B1 present in extrarenal tissues (primarily in immune cells) acquires most of its regulation via calcitriol. The availability of 25‐OHD3 is also under the control of its main binding protein (DBP) and, to a lesser extent, circulating albumin levels [41, 42] (Figure 3).

Moreover, calcitriol can also be catabolized into inactive metabolites under the catalysis of 24‐hydroxylase (CYP24A1) outside renal tissues. The significance of this pathway is valued when CYP24A1 mutations disrupt the regulation of calcitriol [43].

Therefore, the conversion of vitamin D3 into bioactive calcitriol within the immune microenvironment acts as a critical signaling switch. In the following section, we will detail the consequences of this signal, examining how calcitriol reprograms innate immune cells toward an anti‐inflammatory phenotype and simultaneously restrains pathogenic adaptive immunity by influencing T and β‐cell fate.

## 4. Vitamin D in Immunity

Vitamin D is critical for the regulation of innate and adaptive immunity. Given the widespread distribution of its receptor in several immune cell patterns, it is no wonder that it has implications in different roles in these cells. Specifically, it blocks β‐cell maturation, division, and production and secretion of the autoantibodies; hinders the proliferation of T cells; and redirects the predominant anti‐inflammatory phenotype from Th1 to Th2 while suppressing the inflammation‐favoring Th17 pattern in addition to stimulating regulatory T (Treg) cells [44]. At the subcellular level, it has inhibitory and antagonistic actions on the production as well as secretion of the inflammatory cytokines derived from monocytes. Also, vitamin D has been documented to be responsible for dominating the action of immune tolerance mediated by dendritic cells. The good news here is that these effects are local (either autocrine or paracrine) since they are mediated by activation of vitamin D receptors (VDRs). This signifies the autoimmunity inhibition to the B, T, and antigen‐presenting cells accused in inflammation and autoimmunity niche [45]. Infections are associated with low vitamin D levels, increasing the predisposition to opportunistic pathogens. Fortunately, therapeutic intervention with vitamin D supplements proved beneficial in autoimmune patients [46].

The human immune system comprises two main categories: innate and adaptive immune systems. The innate immune system involves the physical barriers (such as gastrointestinal and respiratory epithelium), chemical weapons (such as gastric HCl), and biological commensals (represented by gut microbiome). The role vitamin D plays in this system involves regulating the epithelium tight junctions that connect cells together and govern the programmed cell death of gut epithelial and gland cells [47, 48].

Although vitamin D has been investigated for its role in the innate immune system, most studies have concentrated on how it affects antigen‐presenting cells, including macrophages and dendritic cells. Through the use of pattern recognition receptors (PRRs), these cells are essential for identifying and reacting to pathogen‐associated molecular patterns (PAMPs). 1,25‐(OH)2D3, the active form of vitamin D, binds to the VDR and interacts with DNA sequences known as vitamin D response elements (VDREs) that are present in a number of genes linked to PRR function. These genes include those that code for antibacterial proteins such as TREM‐1, β‐defensin 2 (DEFB4), hepcidin (HAMP), cathelicidin (CAMP), and nonobese diabetic (NOD) 2 [49].

**Figure 4:**
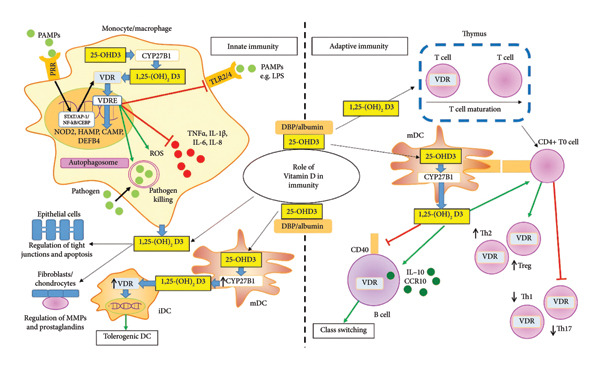
Role of calcitriol in regulating innate as well as adaptive immune responses in autoimmune disorders and its effects on different immune cells therein. The active form of vitamin D, calcitriol, exerts pleiotropic effects on immune cell function. (Left) Innate immune regulation: In monocytes, macrophages, and dendritic cells (DCs), calcitriol signaling enhances antimicrobial defense by upregulating reactive oxygen species (ROS) for pathogen killing. Concurrently, it induces an anti‐inflammatory and tolerogenic phenotype characterized by increased secretion of anti‐inflammatory cytokines (e.g., IL‐10) and decreased production of proinflammatory cytokines (e.g., IL‐12 and IL‐23). This promotes the differentiation of regulatory T cells (Tregs). (Right) Adaptive immune regulation: Calcitriol directly inhibits the proliferation and proinflammatory function of T‐helper 1 (Th1) and T‐helper 17 (Th17) cells. It promotes the development of Tregs, both indirectly via tolerogenic antigen‐presenting cells and directly by influencing T cell maturation and differentiation. In β cells, calcitriol binds to vitamin D response elements (VDREs) to inhibit plasma cell differentiation and promote a regulatory phenotype, thereby dampening autoantibody production. Together, these mechanisms highlight the central role of calcitriol in reestablishing immune homeostasis in autoimmune disorders. Figure was reused from [51] under CC‐BY 4.0 license.

One important subset of PRRs is toll‐like receptors (TLRs). Studies have shown that 1,25‐(OH)2D3 can suppress the expression of TLR2 and TLR4 in monocytes, which can stop TLR‐mediated immune responses to damage‐associated molecular patterns (DAMPs) and PAMPs from being overexpressed. Furthermore, research has demonstrated that single‐nucleotide polymorphisms (SNPs) in the VDR and the methylation of the VDR gene can impact how the VDR affects TLR1/2 signaling in monocytes [50]. Figure 4 elucidates the interplay of vitamin D3 with immune cell components.

Moreover, calcitriol directly affects adaptive immune cells. When thymocytes grow, VDR is momentarily expressed. When peripheral T and β cells face immunological difficulties, VDR is then reexpressed. It is now known that calcitriol plays a more significant role in determining the phenotypes of T cells than it did in the past when it came to controlling the maturation as well as division of T and β cells [34].

In particular, calcitriol has been demonstrated to stimulate the development of Th2 cells and Treg cells while suppressing the activity of inflammatory CD4+ T‐helper (Th) cells, such as Th17 and Th1 cells that produce interleukin‐17 [35]. Nonetheless, vitamin D has a role in strengthening specific immune responses by increasing effector T cell activities, such as CD8+ T cell cytotoxicity. Some investigations have shown that calcitriol upregulates TCR and, thereby, facilitates stimulation and activation of T cells [52].

Calcitriol has also a modulatory action on NK cells and NKT cells, where it has been observed to enhance these cells’ activation and cytotoxicity. Evidence suggested that the maturation of NKT cells was disrupted in VDR knock‐out mice [53].

## 5. Vitamin D and Autoimmunity

The role of vitamin D in immune modulation has garnered significant attention over recent decades, establishing its importance in regulating both innate immunity and acquired (adaptive) immunity. Calcitriol binds and activates VDR to exert various immunological effects, including the suppression of inflammatory processes and the promotion of tolerogenic responses. Recent reports indicate that vitamin D insufficiency is associated with a heightened risk of autoimmune diseases, which exhibit notable sex differences attributed to genetic, epigenetic, hormonal, and environmental factors. Recent studies have highlighted the interplay between vitamin D and estrogen, a sex hormone that can modulate immune responses [54]. Depending on its concentration, estrogen may skew immune responses toward either a Th1 or Th2 profile, thus influencing the pathogenesis of different autoimmune disorders. Notably, estrogen has been shown to enhance the efficacy of vitamin D by promoting its accumulation and increasing VDR expression, leading to a more robust anti‐inflammatory response in females compared to males. Conversely, vitamin D has been observed to downregulate the expression of aromatase in immune cells, an enzyme responsible for converting testosterone to estrogen, which may contribute to decreased estrogen levels [55]. These findings suggest a potentially greater protective effect of vitamin D‐based therapies in women, particularly those of reproductive age, compared to their male counterparts.

Vitamin D is increasingly recognized for its immunomodulatory capabilities, initially noted for its immunostimulatory effects upon discovery [56]. Over time, as the prevalence of autoimmune diseases has risen globally, a corresponding increase in vitamin D deficiency has been documented. This correlation highlights the potential significance of vitamin D in fostering immune tolerance and suggests that its deficiency may play a critical role in the onset of autoimmune disorders [57, 58]. Furthermore, data point to the potential that vitamin D’s impact on autoimmunity has sex‐specific features, especially in relation to connections to estrogen [55, 59].

**Figure 5:**
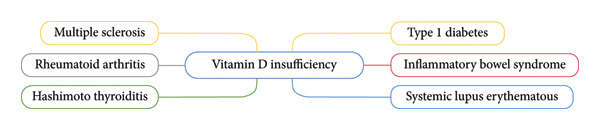
Autoimmune disorders that are characterized by vitamin D deficiency, ranging from Type 1 diabetes to systemic lupus erythematosus.

In addition to rheumatoid arthritis, vitamin D deficiency has been associated with other autoimmune conditions such as systemic lupus erythematosus [60, 61], Hashimoto thyroiditis [62] and multiple sclerosis [63, 64]. Furthermore, individuals with inflammatory bowel disease (IBD) frequently present with low vitamin D levels, which appear to correlate with disease activity. Vitamin D plays a vital role in maintaining the integrity of the intestinal barrier and is essential for microbial balance in the gut [65]. Its anti‐inflammatory properties may help prevent IBD by supporting barrier function, promoting bacterial homeostasis, and mitigating disease progression. Notably, there is evidence suggesting that vitamin D may even induce remission in patients suffering from Crohn’s disease [66]. Autoimmune disorders in which vitamin D is deficient are depicted in Figure 5.

Interestingly, some patients may exhibit a resistance to vitamin D, indicating that a one‐size‐fits‐all approach to treating vitamin D deficiency may not be effective. This observation underscores the need for personalized treatment strategies that consider individual variations in vitamin D metabolism and response.

Low levels of serum 25D are a widespread global health concern. This deficiency poses significant challenges to maintaining calcium balance and bone health in both adults and children. In addition, there has been a notable surge in research highlighting the connection between 25D deficiency and health issues beyond the skeletal system. One of the most prominent findings is the correlation between low serum 25D levels and immune system dysfunction, particularly in relation to autoimmune diseases. A consistent finding across these studies is that reduced serum 25D concentrations are linked to a higher prevalence and/or increased severity of autoimmune conditions. However, a crucial question remains unresolved: Is 25D deficiency a cause or a result of autoimmune disorders.

To explore this question further, recent research has investigated the role of genetic variability within the vitamin D system as an indicator of lifelong differences in 25D levels. One method employed is examining whether SNPs in genes related to vitamin D metabolism, transport, or function are associated with the prevalence or severity of autoimmune diseases. Key genes studied include those encoding serum DBP (GC), 25‐hydroxylase (CYP2R1), CYP27B1, 24‐hydroxylase (CYP24A1), and the VDR. The overall conclusion from these studies suggests that genetic variations, particularly in the VDR gene, may influence susceptibility to autoimmune diseases. However, a major limitation is that the functional significance of many of these SNPs remains unclear, making it difficult to fully understand the impact of genetic variability in the vitamin D system on autoimmune disease risk.

Although vitamin D deficiency is most commonly correlated with autoimmune defects, it is not the sole contributing factor. Albeit rare, vitamin D resistance emerged as another cause of autoimmunity. Indeed, the concept of vitamin D resistance posits a more profound dysfunction at the tissue level. In this model, circulating 25(OH)D levels may be normal or even elevated, but there is a blunted physiological response to the active hormone, calcitriol. This resistance could be driven by several mechanisms intrinsic to T1D pathophysiology. These include downregulation of the VDR in immune cells, impaired expression or activity of the 1α‐hydroxylase (CYP27B1) enzyme needed for local hormone activation, or perturbations in the downstream signaling cascade within target cells. This is analogous to insulin resistance in T2D, where hormone levels are adequate, but cellular responsiveness is diminished [67]. From a clinical perspective, this model explains why simply administering more substrate (oral vitamin D) may be ineffective; the problem lies not in the hormone’s availability, but in the cell’s ability to receive and execute its signal. A state of resistance would necessitate much higher levels of 25(OH)D to saturate the system and overcome the blunted response, or alternatively, therapeutic strategies aimed at sensitizing the VDR pathway.

The immunomodulatory capacity of vitamin D is principally mediated by the genomic binding of its active metabolite, calcitriol, to the vitamin VDR, a nuclear transcription factor expressed in immune cells, which subsequently promotes a state of immune tolerance and suppresses inflammatory pathways. This endocrine‐immune axis is profoundly influenced by sex‐specific factors, creating a bidirectional regulatory network. Estrogen, in particular, potentiates vitamin D signaling by upregulating VDR expression and the activity of the 1α‐hydroxylase (CYP27B1) enzyme, thereby enhancing the anti‐inflammatory and tolerogenic effects of calcitriol in females [68]. Conversely, vitamin D can downregulate the expression of aromatase, the enzyme responsible for converting androgens to estrogen, establishing a critical feedback loop that interconnects vitamin D status with sex hormone bioavailability. This complex crosstalk may provide a mechanistic basis for the observed female predisposition to autoimmune conditions such as T1D, and it further suggests that the therapeutic efficacy of vitamin D supplementation is likely to be sexually dimorphic, with potentially greater protective benefits in reproductive‐aged women [69].

Having established vitamin D’s role as an immunomodulator, we now turn to its specific implications for T1D. The following section will examine how the mechanisms described above, particularly the promotion of T‐regulatory cells and suppression of Th1 responses, are directly relevant to the pathogenesis of T1D, which is driven by the autoimmune destruction of pancreatic β cells.

## 6. T1D and Vitamin D

**Figure 6:**
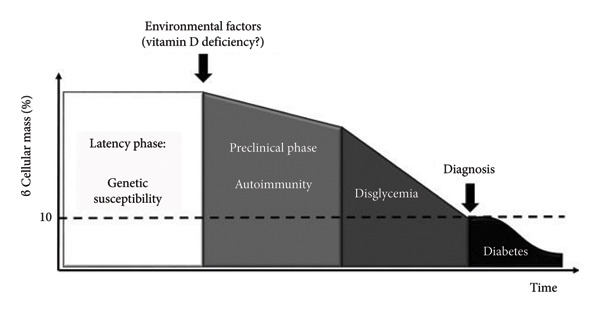
Successive phases of autoimmune Type 1 diabetes pathogenesis (latency phase, preclinical phase, and dysglycemia) that culminate with disease onset. Figure was reused from [74] under CC‐BY 4.0.

T1D is a long‐term autoimmune disorder marked by the targeted and gradual destruction of insulin‐producing β cells in the pancreatic islets, predominantly affecting individuals with a genetic predisposition [70]. However, the relatively low occurrence of T1D among identical twins suggests that environmental factors may significantly contribute to the onset of β‐cell damage and the subsequent development of the disease [71]. The autoimmune basis of T1D is clinically validated by the detection of circulating islet autoantibodies, which serve as key biomarkers for disease prediction and staging. The presence of multiple autoantibodies signifies a high risk of progression to clinical disease. This understanding has led to a new staging classification: Stage 1 (autoimmunity with normoglycemia), Stage 2 (autoimmunity with dysglycemia), and Stage 3 (symptomatic onset) [72] (Figure 6). Recent clinical trials have successfully leveraged this immunological knowledge for prevention. For instance, teplizumab, an anti‐CD3 monoclonal antibody that modulates T cell responses, has been shown to delay the onset of Stage 3 T1D in high‐risk individuals, representing a landmark achievement in immunotherapy for autoimmune diabetes [73].

In an open‐label randomized trial, Baidal et al. [75] explored the relationship between vitamin D levels and the body’s remaining insulin production in individuals newly diagnosed with T1D. The analysis revealed a significant and direct correlation, showing that higher vitamin D levels were strongly associated with higher fasting C‐peptide, a marker of insulin production (*r* = 0.589, *p* = 0.01). Furthermore, when comparing groups, the majority of participants (56%) were vitamin D deficient, and this group demonstrated a significantly lower fasting C‐peptide level compared to those with sufficient vitamin D (0.22 vs. 0.41 pmol/mL, *p* < 0.006). These results suggest that low vitamin D levels at diagnosis may be linked to a more substantial loss of insulin‐producing cells, but the researchers emphasize that larger studies are needed to confirm this connection.

There is growing evidence pointing to the involvement of various environmental agents in T1D, with vitamin D emerging as a key player. In addition to its well‐known role in maintaining calcium balance and bone health, vitamin D is also crucial in modulating both innate and adaptive immune responses, as well as influencing cardiovascular and metabolic processes. This broad influence is due to the widespread expression of VDR in most nucleated cells, including pancreatic β cells, and in tissues sensitive to insulin, such as skeletal muscle, heart muscle, and fat tissue [76, 77].

At the biomolecular level, the positive effects of vitamin D are attributable to the fact that calcitriol, when it binds with VDR, upregulates insulin gene expression as well as its secretion. Besides, it ameliorates apoptosis and oxidative stress of β‐islets of Langerhans [78, 79]. Plus, it activates the PI3K‐AKT‐FOXO1 pathway that has a significant signaling cascade of insulin leading to improvement in insulin sensitivity and plasma glucose maintenance [80]. T1D patients usually suffer from difficulties in controlling plasma glucose. In a pilot study that continuously monitored plasma glucose in these patients, research teams found that vitamin D supplementation greatly limited plasma glucose variability, which mirrored as reduced hypoglycemic events while mitigating high insulin administrations [81].

Recent articles have identified a connection between diminished serum vitamin D levels and various autoimmune diseases, including T1D. Vitamin D plays a role in regulating the expression of more than 200 genes, many of which are involved in immune system function, indicating its potential involvement in the development of these diseases. The ability of vitamin D to influence the immune response and regulate anti‐inflammatory cytokines suggests that the vitamin D system could be a promising target for developing new treatment strategies for autoimmune diseases, particularly T1D [74].

In vitro and in vivo reports have consistently demonstrated the potent anti‐inflammatory effects of vitamin D, targeting key cellular players involved in the pathogenesis of T1D. Cathepsin G (CatG), a protease implicated in the antigen presentation of proinsulin, has been shown to contribute to the activation of CD4+ T cells, a critical step in the autoimmune destruction of islet β cells [82]. Vitamin D supplementation has been found to suppress CatG expression in T1D mice, leading to a reduction in CD4+ T cell activation and improved pancreatic β‐cell function. These findings suggest that vitamin D may exert its therapeutic effects in T1D by modulating the immune microenvironment and preventing the destruction of islet β cells [83].

Environmental factors play a pivotal role in the etiology and progression of T1D. Viral infections, toxins, reactive oxygen species (ROS), and chronic inflammation can trigger endoplasmic reticulum (ER) stress, a cellular response to protein misfolding. The unfolded protein response (UPR), mediated by IRE1, PERK, and ATF6, is initially protective but can become detrimental if ER stress is prolonged [84, 85]. Recent studies have highlighted the significance of the UPR in T1D pathogenesis. Deletion of IRE1α in β cells of NOD mice has been shown to ameliorate islet inflammation and β‐cell apoptosis, suggesting that modulating the UPR may represent a therapeutic strategy. Furthermore, inflammatory signaling within β cells, often driven by NF‐κB, has been implicated in promoting autoimmunity and β‐cell dysfunction in prediabetic NOD mice. These findings underscore the complex interplay between environmental factors, ER stress, and inflammation in the development of T1D [86].

Vitamin D has been also shown to exert antioxidant effects in T1D. Studies have demonstrated its ability to reduce oxidative markers, such as superoxide dismutase (SOD), and increase insulin and C‐peptide levels. Plus, it has been shown that calcitriol, a physiologically active form of vitamin D, can act as a potent antioxidant through inhibiting ROS, replenishing glutathione (GSH), and mitigating the proatherogenic consequences of elevated ketones in cells of the endothelial system [87]. NADPH oxidase (NOX), a key enzyme involved in the production of ROS, has been implicated in β‐cell death [88]. Therefore, targeting oxidative stress through vitamin D supplementation may offer a promising approach to mitigating β‐cell dysfunction and improving glycemic control in T1D.

A recent systematic review in 2022 investigated the relationship between vitamin D and T1D. The findings suggest a strong association between low vitamin D levels and T1D risk, particularly in early life. While animal studies demonstrate the benefits of vitamin D supplementation, human trials have yielded mixed results. However, there is evidence that maintaining optimal circulating levels of vitamin D may reduce T1D risk and delay the development of absolute or near‐absolute C‐peptide deficiency. Given the low toxicity of vitamin D, daily supplementation is recommended for individuals with T1D or at high risk of developing the condition, especially those with vitamin D insufficiency [14].

Numerous clinical trials as well as intervention studies have examined the outcome of vitamin D supplementation on diabetic and immunologic measurements with conflicting findings.

In 2017, a controlled clinical trial investigated the efficacy of a 12‐week, high‐dose oral cholecalciferol supplementation (2000 IU/day) in correcting vitamin D deficiency and improving glycemic control among 103 children and adolescents with established T1D. The intervention was highly successful in rectifying the underlying biochemical deficiency, demonstrating a large effect size (Glass’s Δ = 1.2) by elevating mean serum 25(OH)D concentrations from 19.2 ng/mL to 30.9 ng/mL. However, this robust normalization of vitamin D status translated to only a minimal and nonsignificant effect on the primary metabolic outcome of glycemic control (Glass’s Δ = 0.1 for HbA1c). Subsequent analysis identified that higher insulin dose and body mass index were negative predictors of postsupplementation vitamin D levels, while sedentary behavior was significantly associated with higher HbA1c, suggesting that these lifestyle and clinical factors may exert a more substantial influence on glycemic outcomes than vitamin D repletion alone in this pediatric T1D cohort [89].

Findings from a 3‐year RCT indicate that pharmacological supplementation with ergocalciferol may decelerate the natural history of glycemic deterioration in T1D. Adolescents receiving a weekly dose of 50,000 IU for 36 months displayed a significantly attenuated rate of HbA1c increase relative to controls. This result implies that sustained vitamin D repletion could modulate underlying pathophysiological processes, such as chronic inflammation or residual β‐cell stress, thereby favorably altering the trajectory of glycemic control in a young T1D population [90].

Another 20‐month, open‐label, RCT evaluated the efficacy of a multipronged immunomodulatory strategy in patients aged 12 years and older with T1D. The intervention arm received a regimen of etanercept (a TNF‐α inhibitor), GAD‐alum (an autoantigen vaccine), and daily adjunctive calciferol (2000 IU). The primary outcome, measured by stimulated C‐peptide, showed a statistically significant interim benefit at 6 months in the treatment group, pointing to an initial positive interaction between the components. Despite this early promise, the final analysis revealed that the intervention failed to achieve its primary endpoint, with no statistically significant difference in C‐peptide preservation, glycemic parameters, or insulin dose sustained over the full 20‐month study period, highlighting the transient nature of the observed effect [91].

Of the 133 pediatric participants with T1D screened in a controlled clinical trial, the 103 individuals (77.4%) identified as vitamin D deficient (25(OH)D < 30 ng/mL) received oral cholecalciferol supplementation at 2000 IU/day for 12 weeks. The intervention was highly effective in correcting the underlying deficiency, significantly elevating serum 25(OH)D concentrations from 19.2 ± 6.2 ng/mL to 30.9 ± 10.1 ng/mL (Glass’s Δ = 1.2). However, this robust biochemical correction translated to only a minimal effect on glycemic control (Glass’s Δ = 0.1 for HbA1c). The study further identified that a higher insulin dose and elevated body mass index were associated with lower postintervention vitamin D levels, while a sedentary lifestyle was significantly correlated with higher HbA1c, suggesting that these modifiable factors may exert a more substantial influence on metabolic outcomes than vitamin D repletion alone in this population [92].

Table 1 summarizes the latest clinical trials using vitamin D supplementation for intervention in T1D.

**Table 1:** Recent clinical trials testing the efficacy of vitamin D3 supplementation for Type 1 diabetics, clarifying the sample size, intervention, dose, duration, and the corresponding key outcomes.

Clinical trial setting	Sample size	Intervention and dose	Duration	Key outcomes	References
Controlled clinical trial	103 Children and adolescents with T1D and vitamin D deficiency	Cholecalciferol (vitamin D3) 2000 IU/day	12 weeks	• Robust correction of vitamin D deficiency: Serum 25(OH)D increased from 19.2 to 30.9 ng/mL (large effect size, Glass’s Δ = 1.2) • Minimal effect on glycemic control: nonsignificant change in HbA1c (small effect size, Glass’s Δ = 0.1) • Negative predictors: higher insulin dose and BMI predicted lower postsupplementation vitamin D levels • Lifestyle factor: sedentary behavior was associated with higher HbA1c	[89]

Randomized controlled trial (RCT)	Adolescents with T1D (specific size not stated)	Ergocalciferol (vitamin D2) 50,000 IU/week	3 years (36 months)	• Slowed glycemic deterioration: significantly attenuated rate of HbA1c increase compared to control • Suggests a disease‐modifying effect: indicates that sustained vitamin D repletion may slow the underlying disease progression	[90]

Open‐label RCT	Patients aged ≥ 12 years with T1D (specific size not stated)	Calciferol (vitamin D) 2000 IU/day *Plus:* etanercept and GAD‐alum	20 months	• Transient benefit: significant interim benefit in stimulated C‐peptide at 6 months • No sustained effect: failed to achieve primary endpoint; no significant difference in C‐peptide preservation, glycemic parameters, or insulin dose over the full 20‐month period	[91]

Controlled clinical trial	133 T1D Pediatrics	Oral cholecalciferol (vitamin D3) at 2000 IU/day	3 months	Primary outcome: • Highly effective correction of vitamin D deficiency • Serum 25(OH)D increased from 19.2 ± 6.2 ng/mL to 30.9 ± 10.1 ng/mL (Glass’s Δ = 1.2) Glycemic outcome: • Minimal improvement in glycemic control (Glass’s Δ = 0.1 for HbA1c) Additional findings: • Higher insulin dose and elevated BMI were associated with lower postintervention vitamin D levels • A sedentary lifestyle was significantly correlated with higher HbA1c	[92]

Despite the promising findings in animal studies, the variable results from human trials of vitamin D in T1D have been somewhat disappointing. These discrepancies can likely be attributed to factors such as the specific form of vitamin D used (25D vs. 1,25D), the duration of intervention, the dosage, and the stage of disease progression. Notably, studies utilizing 25D, the predominant circulating form of vitamin D, have generally shown beneficial effects, while trials with 1,25D, the active metabolite, have often yielded less favorable outcomes. This suggests that increasing circulating 25D levels may be more effective in promoting β‐cell survival in T1D. Given that pancreatic β cells can convert 25D to 1,25D locally, enhancing 25D levels could potentially stimulate the production of 1,25D within the islets, leading to autocrine or paracrine effects [14].

There have been significant strides in guidance regarding efficacy and safety. Past guidelines have commonly recommended daily doses within a range of 800–2000 IU, though studies concluded that 2000 IU/day adequately normalized serum 25(OH)D, while higher doses better maintained such levels [93]. Another well‐designed study has compared the regular daily intake of 2000 IU with a monthly dose of 50,000 IU. It turned out that the latter dose raised the plasma levels of vitamin D more rapidly with no mentioned toxicity [94]. Current recommendations advocate for supplementation at 2000 IU (50 μg) daily among adults to achieve optimal serum levels of at least 75 nmol/L (30 ng/mL) because evidence to support such a dosage has been shown to be safe and effective without adverse effects [95].

Another issue is not considering the insulin resistance in T1D patients. Understanding the link between vitamin D and insulin resistance in T1D is complicated by a fundamental challenge: how to measure insulin resistance itself. Since people with T1D do not produce their own insulin, resistance is diagnosed by showing a weakened response to injected insulin. The gold‐standard method for this is the euglycemic hyperinsulinemic clamp, a rigorous but complex procedure. This measurement hurdle makes it difficult to clearly establish what role, if any, vitamin D plays [96]. The possible link between vitamin D deficiency and insulin resistance in T1D is obesity, which has been shown to be currently a frequent scenario [97, 98].

This shift reflects the broader view of vitamin D on health, placing a higher serum level as important for overall health.

## 7. Combination Therapy

It should be noted that when we assess vitamin D status, 25‐OHD3 is what is measured and, hence, extrarenal production of calcitriol will affect blood levels of 25‐OHD3. This means that for vitamin D to exert its antiautoimmunity effects, it should be ingested in extremely high doses to satisfy the immune‐related and immune‐unrelated actions. In addition, combining vitamin D administration with other medications showed a substantially beneficial impact on T1D patients.

For example, L‐cysteine when co‐ingested with vitamin D enhanced the metabolic action of vitamin D by mitigating inflammation and supporting the expression of key vitamin D‐regulating genes, thereby offering a promising therapeutic strategy for addressing vitamin D deficiency and its associated health risks in populations at higher risk, such as those with metabolic syndrome and diabesity [99].

Likewise, another work explored the synergistic effects of low doses of anti‐CD3 monoclonal antibodies, ciclosporin A, and the vitamin D analog TX527 in delaying the recurrence of autoimmune diabetes in a NOD mouse model following islet transplantation. Researchers concluded that while anti‐CD3 therapy has shown promise in treating recent‐onset T1D, its efficacy is limited by a narrow therapeutic window. The combination with ciclosporin A and TX527 enhances immunomodulatory effects, promoting the induction of Treg cells and improving the preservation of β‐cell function. The findings suggest that this multiagent approach could offer a more effective strategy for preventing diabetes recurrence in islet‐transplanted patients, paving the way for potential clinical applications in autoimmune diabetes management [100].

Moreover, another report examined the potential of combining vitamin D with dipeptidyl peptidase‐4 inhibitors (DPP‐4i) as an immunomodulatory therapy for autoimmune diabetes, specifically in T1D. It turned out that the combination improved the preservation of β‐cell function and prolonged the “honeymoon phase” in newly diagnosed T1D patients by exerting synergistic anti‐inflammatory and immunomodulatory effects. Preclinical and preliminary clinical data suggest that this combination therapy could counteract β‐cell autoimmunity and maintain β‐cell mass, thus offering a promising therapeutic strategy for managing autoimmune diabetes [101].

Even in the case of latent autoimmune diabetes in adults (LADA), which is marked by positive antiglutamic acid decarboxylase antibodies and poor glycemic control. Following the diagnosis, the patient was treated with a combination of sitagliptin, a dipeptidyl peptidase‐4 inhibitor, and vitamin D supplementation due to an identified deficiency. Remarkably, this combined treatment led to a significant decrease in GAD‐abs levels, normalization of antibody status, and improved glycemic control, with HbA1c levels dropping from 9.6% at diagnosis to 5.2% after 2 years without the need for insulin. The findings suggest that the combination of sitagliptin and vitamin D may effectively modulate autoimmune responses and preserve β‐cell function in LADA patients, indicating a potential therapeutic strategy for managing this form of diabetes [102].

More recently, Yan et al. [103] conducted a multicenter RCT for testing the combination efficacy of saxagliptin and cholecalciferol in keeping β‐cell function in adult‐onset T1D. It turned out that the treatment group displayed C‐peptide concentration in comparison with the control (−276 pmol/L vs. −419 pmol/L; *p* = 0.01). The highest potency was evident in elevated anti‐GADA titers. The adjunctive use of saxagliptin enabled a decrease in insulin requirements while achieving a similar level of glycemic control.

This evidence emphasizes the efficacy of coupling therapy with vitamin D in greatly alleviating the incidence as well as progression of both types of diabetes, possibly through modulating the autoimmune response and boosting the hypoglycemic effect of these drugs.

## 8. Conclusion and Future Prospects

In conclusion, this review has highlighted the intricate relationship between vitamin D and autoimmunity, with a particular emphasis on T1D. While numerous studies have linked vitamin D deficiency to many autoimmune illnesses, the underlying mechanism that clearly fills the gap between vitamin D involvement, in particular in terms of cause or consequence, is yet to be fully identified. The evidence presented in this review suggests that vitamin D plays a crucial role in regulating immune function and may be involved in the pathogenesis of autoimmune diseases. The clinical trials’ findings suffer from low‐dosing and using low active formulas, which need to be addressed in future studies. Further research is needed to fully understand the complex interplay between vitamin D, the immune system, and autoimmune disorders, with the goal of developing effective therapeutic interventions. In addition, clinical trials testing direct sun exposure as a means to get enough and a natural source for vitamin D3 should be found and funded. Finally, coupled or combined therapy achieved unprecedented success in various patterns of autoimmune diabetes, which should have its way in the clinical trial context.

## Ethics Statement

The author has nothing to report.

## Consent

The author has nothing to report.

## Conflicts of Interest

The author declares no conflicts of interest.

## Author Contributions

Conceptualization, data curation, resources, investigation, drafting and editing the manuscript: Haitham Al‐Madhagi.

## Funding

This paper received no funding.

## Data Availability

No data were used nor generated in this study.
